# Laser treatment of 13 benign oral vascular lesions by three different surgical techniques

**DOI:** 10.4317/medoral.18156

**Published:** 2013-02-05

**Authors:** Umberto Romeo, Alessandro Del Vecchio, Claudia Russo, Palaia Gaspare, Gianfranco Gaimari, Josep Arnabat-Dominguez, Antoni J. España

**Affiliations:** 1 DDS. “Sapienza” University of Rome, Department of Oral and Maxillofacial Sciences; 2 DDS, PhD. “Sapienza” University of Rome, Department of Oral and Maxillofacial Sciences; 3 DDS, MD, PhD. University of Barcelona, Dental School

## Abstract

Objectives: Benign Oral Vascular Lesions (BOVLs) are a group of vascular diseases characterized by congenital, inflammatory or neoplastic vascular dilations clinically evidenced as more or less wide masses of commonly dark bluish color. If traumatized BOVLs are characterized by a great risk of hemorrhage and their treatment usually requires great caution to prevent massive bleeding. In the last decades lasers have dramatically changed the way of treatment of BOVLs permitting the application of even peculiar techniques that gave interesting advantages in their management reducing hemorrhage risks. The aim of this study was to evaluate the capabilities and disadvantages of three laser assisted techniques in the management of BOVLs. Study design: In this study 13 BOVLs were treated by three different laser techniques: the traditional excisional biopsy (EB), and two less invasive techniques, the transmucosal thermocoagulation (TMT) and the intralesional photocoagulation (ILP). Two different laser devices were adopted in the study: a KTP laser (DEKA, Florence, Italy, 532nm) and a GaAlAs laser (Laser Innovation, Castelgandolfo, Italy, 808nm) selected since their great effectiveness on hemoglobin. Results: In each case, lasers permitted safe treatments of BOVLs without hemorrhages, both during the intervention and in the post-operative period. The minimally invasive techniques (TMT and ILP) permitted even the safe resolution of big lesions without tissue loss. Conclusions: Laser devices confirm to be the gold standard in BOVLs treatment, permitting even the introduction of minimal invasive surgery principles and reducing the risks of hemorrhage typical of these neoplasms. As usual in laser surgery, it is necessary a clear knowledge of the devices and of the laser-tissue interaction to optimize the results reducing risks and disadvantages.

** Key words:**Oral vascular diseases, laser, photocoagulation.

## Introduction

The Benign Oral Vascular Lesions (BOVLs), are a group of common benign oral neoplasms including haemangiomas and vascular malformations. Vascular lesions often involve the skin and the mucous membranes, but they are also frequent in the mouth and tongue (1). The congenital variant of haemangiomas is often found in the labial mucosa.

Since their content rich in blood and vessels BOVLs are all characterized by high risk of hemorrhage and their treatment needs great attention if performed with traditional surgical techniques. Recently, the use of laser energy as a therapeutic option offered a more conservative, but still effective, approach in their treatment (2). In the study, three different laser approaches of BOVLs are examined: excisional biopsy (EB), transmucosal thermocoagulation (TMT) and intralesional photocoagulation (ILP).

The EB is based on the removal of the whole lesion through a peripheral incision. It is used for small lesions but it may be adopted also in large base lesions. Its disadvantages are mainly bleeding risks, possibility of scars formation especially in aesthetic areas but it is overall less invasive than other traditional blade techniques determining less tissue loss.

The TMT is based on the laser irradiation without contact of the fiber with the tissue. The preferable distance from the lesion surface is about 2-3mm. Energy must be applied with a scanning movement without keeping the fiber fixed on the same point for more than 5-10 seconds to avoid irreversible thermal effects. During the treatment the lesion becomes lighter and smaller. This effect is called “forced dehydration” (3), and it is due to the high absorption of laser energy by the blood into the lesion.

To a more safe execution of the TMT a transparent glass slide is put over the lesion to reduce lesion thickness and to facilitate the laser action. In case of larger lesions, more laser applications may be necessary. The advantages of TMT are: no bleeding risk, no suture needed, bloodless operative field, relative facility and speed of execution, only surface and less invasive anesthesia. The possible necessity of multiple laser sessions and the impossibility of histological examination of the lesion are the main TMT disadvantages. TMT is really helpful if patients are affected by systemic pathologies such as factor VIII of coagulation deficiency. In these cases this no bleeding, safe and quick treatment permits a good resolution of the pathology without any risk, during and after the treatment.

The ILP treatment is another minimally invasive procedure (4). It allows the laser to release its energy directly into the lesions. The main risk related to the technique is that it is a “blind” procedure so a great attention must be dedicated to the exact extent of the lesion to minimize the risk of peripheral tissues damages. The real position of the fiber can be perceived by the transillumination produced by laser. This treatment is useful in deep and large lesions as vascular malformations of the tongue and big hae-mangiomas.

In these cases the KTP laser is the most effective device because of its high specificity for the hemoglobin present in each of these lesions (5-8). Color changes, visible wrinkling and increased hardness of tissue are the signals of the end of the treatment. After the ILP treatment the application of ice over the treated area is helpful to prevent postoperative swelling. ILP therapy allows safe treatment of bulky vascular lesions that cannot be treated by EB because of the danger of massive bleeding or for the treatment of lesions located in aesthetic areas that could create large unaesthetic scarring (5) if removed by traditional methods. Even if ILP must be considered a generally safe technique, intraoperative bleeding risk and impossibility of histological examination are its main disadvantages.

The aim of the study is to evaluate advantages and disadvantages of these techniques and compare their ways of action.

## Material and Methods

Thirteen patients affected by BOVLs ( Table 1) were treated using different laser techniques, by two laser devices: a KTP (DEKA, Florence, Italy, 532nm) and a diode GaAlAs (Laser Innovation, Castelgandolfo, Italy, 808nm). These devices were selected because of their affinity to the oxyhaemoglobin that provokes a photothermolysis, with erythrocytes microagglutination, that produces the vessels obliteration reducing hemorrhage risks. Moreover other advantages permitted by lasers are: reduced necessity of anesthesia, faster healing, more precise cutting, less postoperative discomfort due to the biostimulative effect.

**Table 1:**
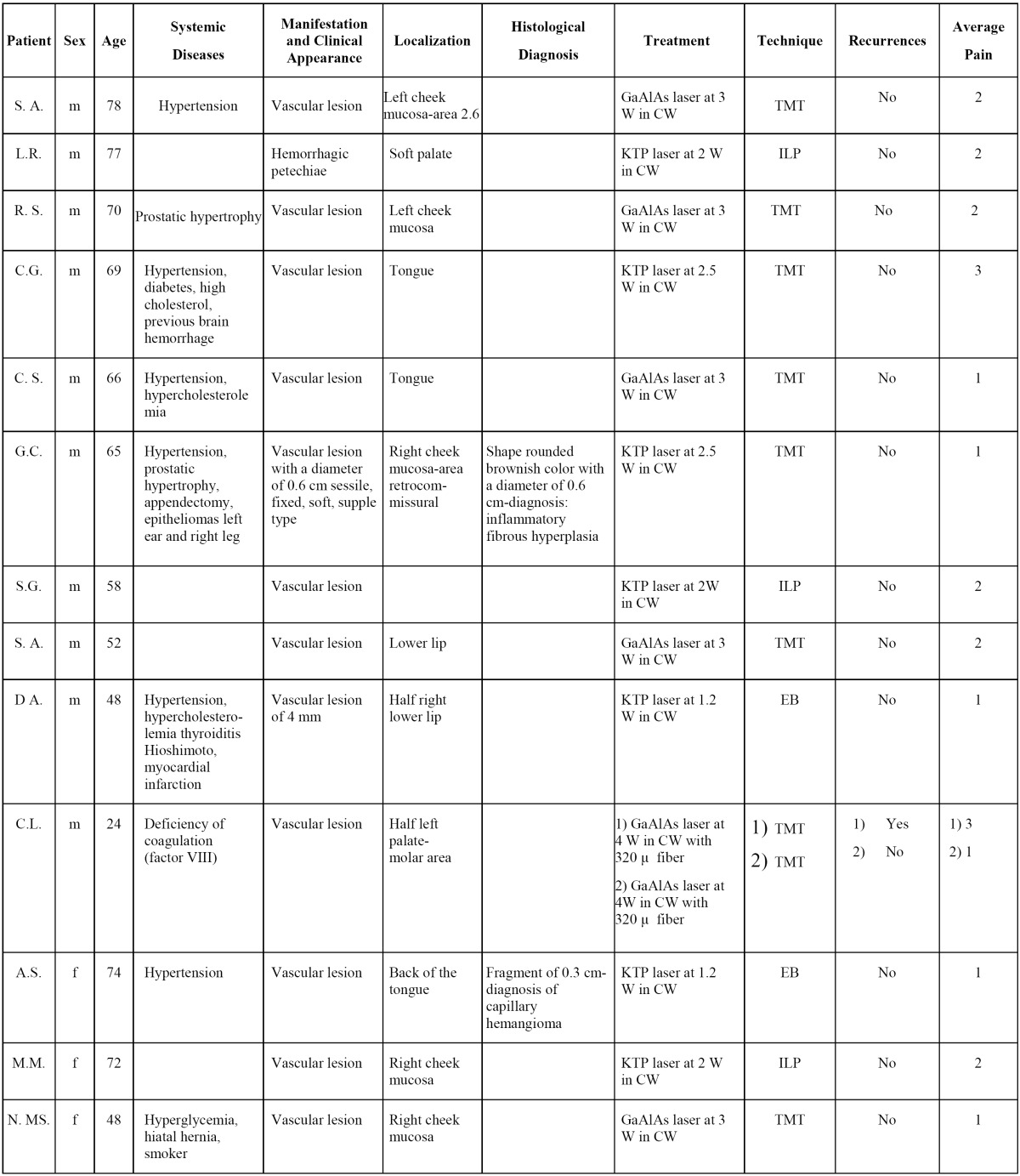
Table resuming patients, kind of lesion and laser treatment.

**Figure 1:**
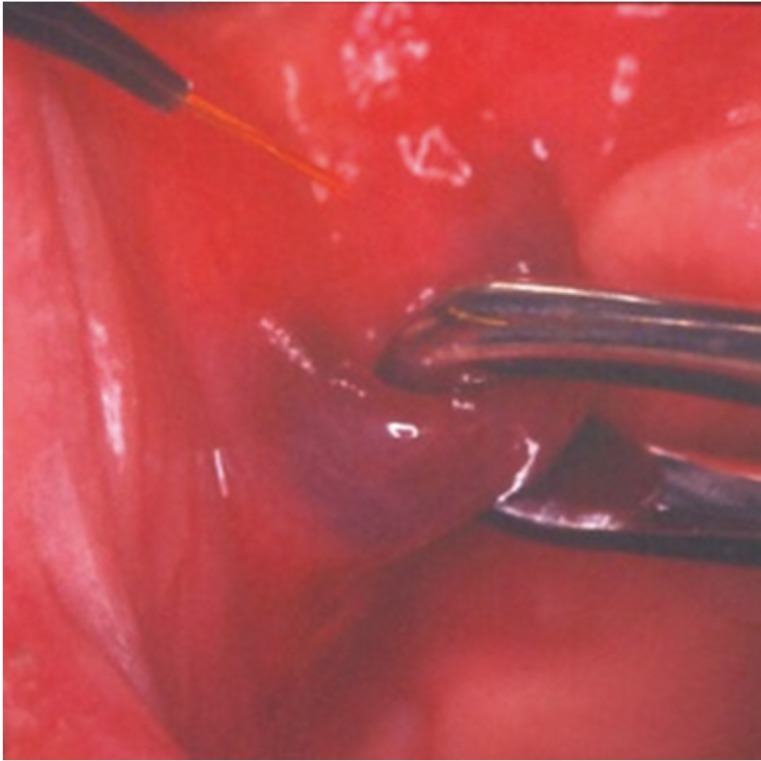
Lesion immobilization by an Allis’ clamp and laser set at 1.2W in CW (EB technique).

Two cases were treated using an EB by a KTP (532nm) laser. The local anesthesia was performed without adrenaline, the choice of this kind of anesthesia is to avoid that the vasoconstriction induced by adrenaline may reduce the laser cut effectiveness. The lesion was immobilized by an Allis clamp and then the excision was performed by a circumferential cut at 1.2W power in continuous wave (Fig. 1).

**Figure 2:**
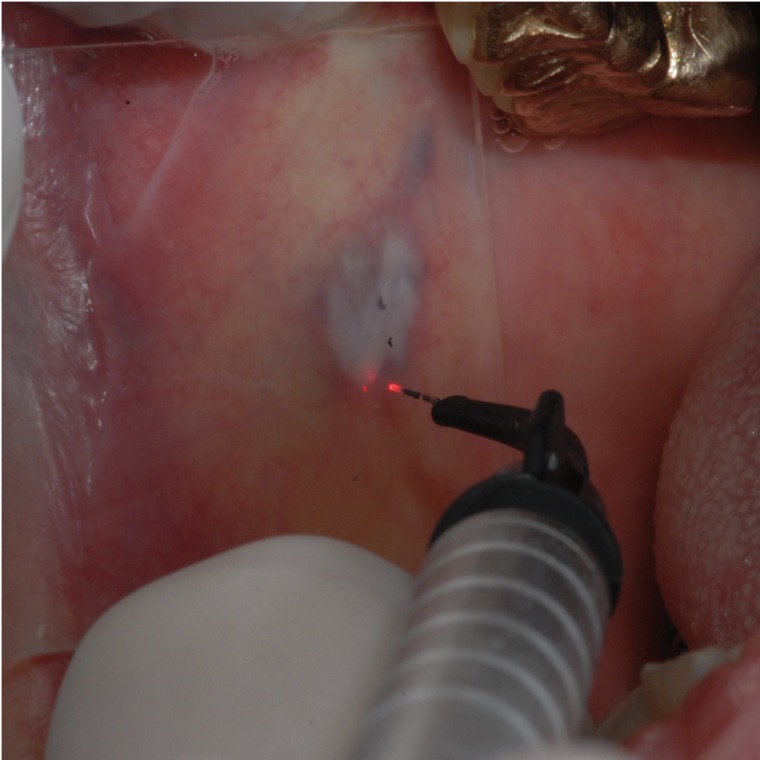
Thin glass put over the lesion (TMT technique).

Eight of the patients were treated by the TMT technique with a KTP (532nm) laser set at 2.5W in continuous wave. After local surface anesthesia by lydocaine cream for 10 minutes, a thin glass was put over the lesion. The irradiation led to an ischemic area (Fig. 2).

A diode GaAlAs (808 nm) laser was used only in one of the TMT cases. It was set at 4W in continuous wave with a 320µ fiber. In this case a second laser application was performed to the complete dehydration of the lesion.

**Figure 3:**
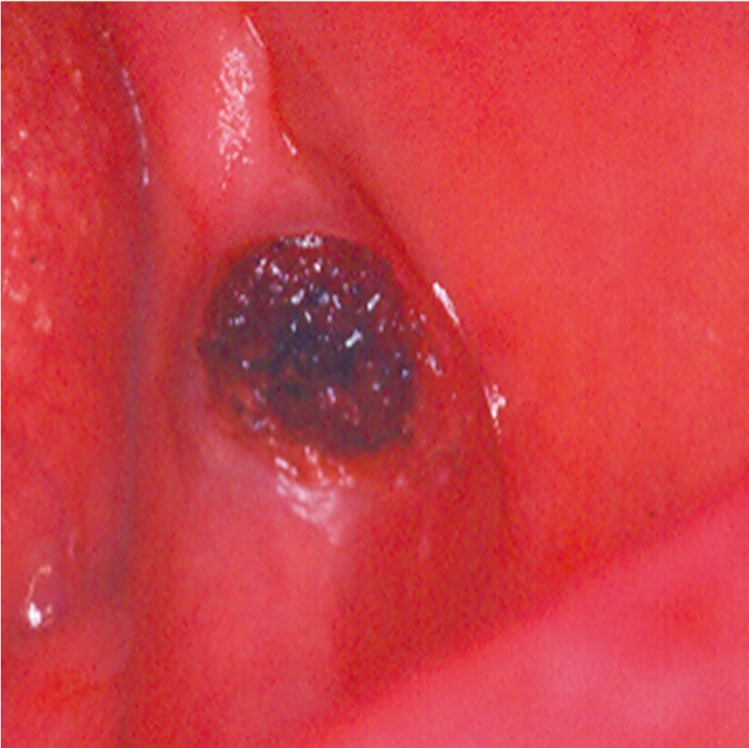
The surface of the vascular lesion after the ILP technique. Laser irradiation at 2W in CW lead to an ischemic area (ILP technique).

The ILP treatment was adopted for the other three patients. In these cases the KTP (532nm) laser at 2W in CW was adopted. After surface lydocaine cream application for 10 minutes, the fiber was put into the lesion provoking its collapse and carbonization (Fig. 3). The fiber was kept into the lesion until its breakdown.

After each treatment, it was asked the patients to evaluate the pain following a Numeric Rating Scale (NRS) in which a value corresponded to 0 indicated the absence of pain instead of a value such as 10 corresponded to the higher and most intensive pain felt in the postoperative period.

## Results

In every case, postoperative tissue sloughing occurred within few days. All the patients healed completely by re-epithelialization within 1 or 2 weeks. No patient referred postoperative bleeding or intensive pain ( Table 1). There were no infections, hematomas or damage to vital structures and ulcerations caused by the heat.

Recurrence happened only in a case in which a TMT technique was used. However, the patient was treated again with the same laser technique and same laser settings and a complete healing was obtained.

## Discussion

Maxillofacial BOVLs are a wide group of pathologies including haemangiomas and vascular malformations (9). They are characterized by morphological, structural and functional alterations of different nature, gravity and extension and may involve every kind of vessel. More than 50% of BOVLs originates from blood vessels or vascular structures. They are frequently located in the head and neck region (8,10).

The right incidence in the population is unknown, even if a progressive increase during the last decades has been described. They are above all sporadic forms found in patients with a negative medical history. However, hereditary forms are described.

The incidence of haemangiomas is between 4-10% in children. In the Tasnàdi series, amongst 3573 children aged 3 years, the incidence of BOVLs was about 1.2% (5).

It is believed that BOVLs arise from two distinct pathways. The first one is the so-called primary vascular malformation. In such situation dysplastic blood vessels tend to drain into adjacent veins. The defect enlarges due to the increase of hydrostatic pressure. According to the second pathway a cavernous haemangioma is formed due to a faulty angiogenesis (a so-called biologic mechanism), which regresses with time (7).

The tumors of blood vessels are a wide group of diseases and they can be divided in benign, malignant and intermediate. Most of these tumors are the result of the differentiation of endothelial cells.

According to ISSVA (International Society for the Study of Vascular Anomalies) of 1992 vascular lesions may be classified in: tumors and malformations ( Table 2).

**Table 2:**
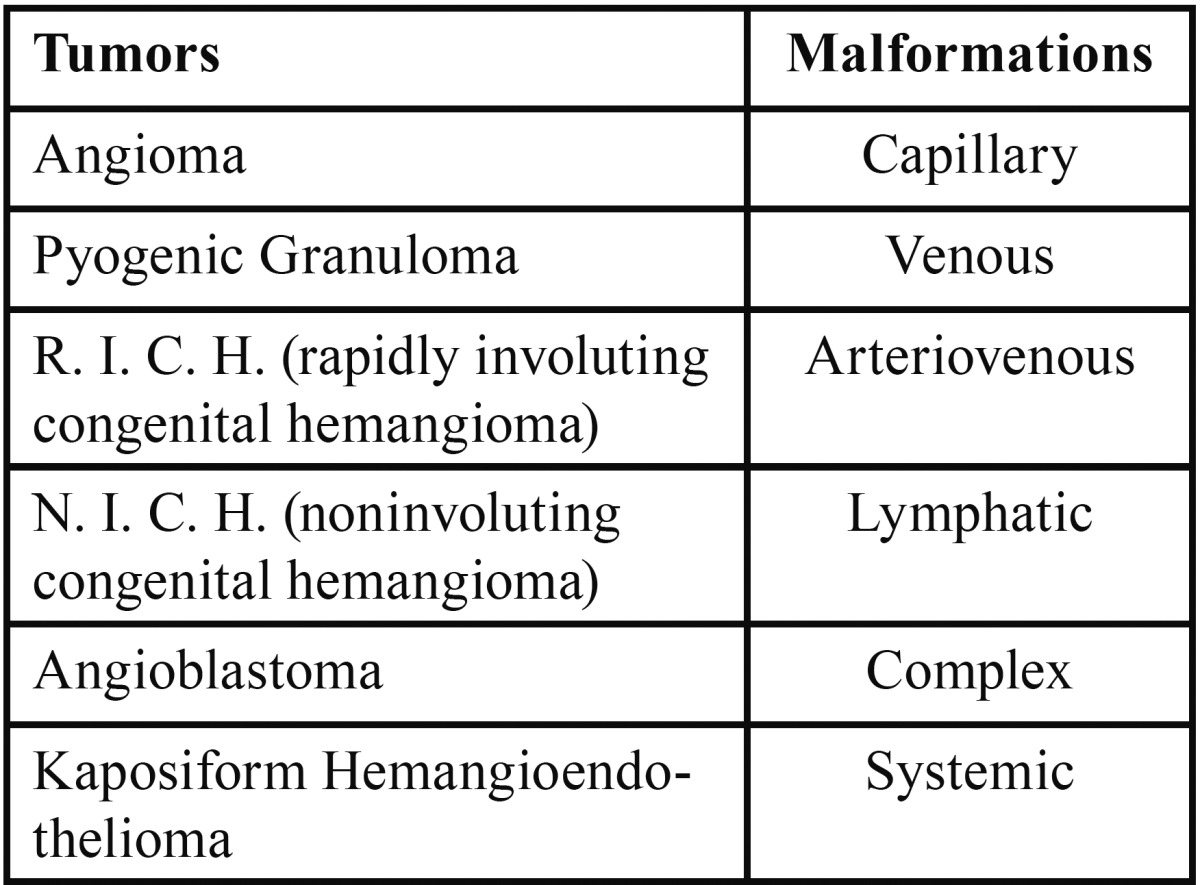
Classification of BOVLs.

In the Literature many other classifications are in use as the Mulliken and Glowacki’s one that is based on cellular criteria. In this sense BOVLs are subdivided in those ones characterized by endothelial proliferation (haemangiomas) and in those ones with normal endothelial turnover (vascular malformations) (11).

The diagnosis of BOVLs is generally based upon several clinical items: color, site, surface, margins, consistence and size.

Haemangiomas are benign tumors of the capillary endothelium very frequent in children. With respect to gender, the literature shows a higher incidence in girls (12-14). They grow quickly during the first weeks of life, but their size remains the same until the age of 12-18 months. Usually they spontaneously diminish in size and their complete self-resolution at the age of 5 years is frequent (15,16). Haemangiomas may be located superficially or deeply everywhere in the oral cavity, more often on the lips and less frequently in the tongue and buccal mucosa (12). They are usually solitary lesions (80%), while multiple lesions (more than three) are often associated to a systemic involvement. Clinically, haemangiomas appear as red patches with irregular but well lined edges. They have a firm, rubbery consistency (11); during the growing phase, high concentrations of type IV collagenase, VEGF, bFGF and urokinase may be histological evidences (17). Haemangiomas complications are necrosis, ulcerations, infections and hemorrhages due to high blood content. In case of large lesions they can also cause difficulty in breathing, in chewing and in speech (12).

Venous and capillary-venous malformations are usually present at birth and grow contemporarily to the child. They are cold and soft and easily compressible (18-21). The overlying mucosa may show a bluish color. Venous malformations are usually solitary lesions. They may expand after local trauma or hormonal changes. Adjacent bony structures are often deformed and invasion of adjacent muscles is common (11).

Arterio-venous malformations are uncommon on the head and neck region. They are evident early after birth and grow slowly with outbreaks linked to hormonal or mechanical factors. They have similar clinical features to the ones observed in port-wine stains, but in these cases the overlying skin is warmer (22-24). Clinically they appear as swellings with different dimensions and extension. A biphasic clinical course is typical. An asymptomatic phase is generally followed by a growing one. During the latter, ulcers of the overlying skin and hemorrhage may occur and adjacent bony structures may be necrotized (11).

Capillary malformations (port-wine stains) are often located in the territory of the branches of the trigeminal nerve. They are characterized by the presence of a thick system of venules and post-capillaries abnormally dilated. Usually, they do not undergo spontaneous involution like haemangiomas (18,19). Clinically they appear as pink or red-purple mucous patches, with clear or jagged margins. Their dimension and extensions are variable. They may be solitary, multiple or confluent. Skeletal hypertrophy and hyperkeratinisation of the overlying skin are common in large lesions. Capillary malformations may be found in association with generalized angiomatosis, such as Sturge–Weber syndrome (11). Furthermore, typical of these lesions is the localization only by one side of the body.

Digital pressure applied over every BOVLs induces ischaemia to the overlying mucosa; this evidence is pathognomonic of the vascular origin of the lesion.

Regarding treatment, corticosteroids have been used to reduce the size of the lesion (11,13), embolization, and intralesional injec-tions of sclerosing agents (25). According to McHeik et al. (13), intralesional injection of corticosteroids may produce ulceration of the lesion. These same authors obtained very good results with surgical treatment of haemangiomas at an early age in cases with aesthetic problems or complications such as ulceration, pain, bleeding and infection. In a study of Minguez-Sanz et al., 13 were surgically removed, 2 were treated by embolization, and 13 disappeared spontaneously. Most congenital haemangiomas in this study regressed spontaneously without requiring treatment.

In the last decades lasers greatly enhanced both treatment and prognosis of BOVLs; these devices may avoid many complications related to these pathologies, in particular as the hemorrhages, typical of the conventional surgery. Laser allows an easy removal, even if its use is not safe in arterial lesions. In these latter the embolization of the main artery is mandatory.

The interaction between laser and tissue is due to the energy absorbed by tissues. For the correct treatment of a lesion with high blood content it is necessary to choose a laser emitting in a wavelength well absorbed by haemoglobin. KTP, diode, Nd:YAG and CO2 are the most effective wavelengths in these cases (4,23,26).

Moreover for a correct laser treatment of BOVLs it is necessary to well evaluate many further parameters as: pulse duration, spot size and the energy density. The choice of the pulse duration should be done according to the size of the vessels to be treated. Shorter pulse durations are preferable for small diameter vessels while longer pulse durations must be employed in larger diameter ones. The spot size selection should be based according to the depth and the size of the lesion. Finally, the selection of the energy density should be based on the color of the lesion, purple and bluish lesions absorb laser energy more than pink or red ones requiring for this reason lower fluencies (2).

According to our experience it is possible to affirm that lasers are the gold standards in the treatment of BOVLs of the oral cavity with venous flow. The EB, the sole to permit a histological diagnosis of the lesion must be reserved to vascular lesions suspected to be malignant neoplasms.

Amongst the laser techniques the TMT seems to be the most reliable and advantageous. In fact, in TMT the optical fiber is not in contact with the lesion, resulting so absolutely safe and sure even in patients affected by systemic diseases as coagulation problems or in anticoagulant therapies.

 This method permits also good functional and aesthetic results and it gives poor or no intra- and postoperative complications. Its sole contraindication is the impossibility to perform the histological examination.

The ILP is often used for wide and deeper lesions that cannot be treated with TMT. ILP is characterized by a higher risk of bleeding due to the fiber penetration into the lesion, but it can be overall considered a safe technique.

It is possible to assess that laser treatment of BOVLs is safe and effective, and in many cases it represents the gold standard technique permitting results unattainable with conventional treatments.
